# Positive selection and intrinsic disorder are associated with multifunctional C4(AC4) proteins and geminivirus diversification

**DOI:** 10.1038/s41598-021-90557-0

**Published:** 2021-05-27

**Authors:** Carl Michael Deom, Marin Talbot Brewer, Paul M. Severns

**Affiliations:** Department of Plant Pathology, University of Georgia, Athens, GA 30602 USA

**Keywords:** Microbiology, Molecular biology, Evolution, Evolutionary genetics, Phylogenetics, Population genetics

## Abstract

Viruses within the *Geminiviridae* family cause extensive agricultural losses. Members of four genera of geminiviruses contain a *C4* gene (*AC4* in geminiviruses with bipartite genomes). *C4(AC4)* genes are entirely overprinted on the *C1(AC1)* genes, which encode the replication-associated proteins. The C4(AC4) proteins exhibit diverse functions that may be important for geminivirus diversification. In this study, the influence of natural selection on the evolutionary diversity of 211 *C4(AC4)* genes relative to the *C1(AC1)* sequences they overlap was determined from isolates of the *Begomovirus* and *Curtovirus* genera. The ratio of nonsynonymous (*d*_N_) to synonymous (*d*_S_) nucleotide substitutions indicated that *C4(AC4)* genes are under positive selection, while the overlapped *C1(AC1)* sequences are under purifying selection. Ninety-one of 200 *Begomovirus C4(AC4)* genes encode elongated proteins with the extended regions being under neutral selection. *C4(AC4)* genes from begomoviruses isolated from tomato from native versus exotic regions were under similar levels of positive selection. Analysis of protein structure suggests that C4(AC4) proteins are entirely intrinsically disordered. Our data suggest that non-synonymous mutations and mutations that increase the length of C4(AC4) drive protein diversity that is intrinsically disordered, which could explain C4/AC4 functional variation and contribute to both geminivirus diversification and host jumping.

## Introduction

**Figure 1:**
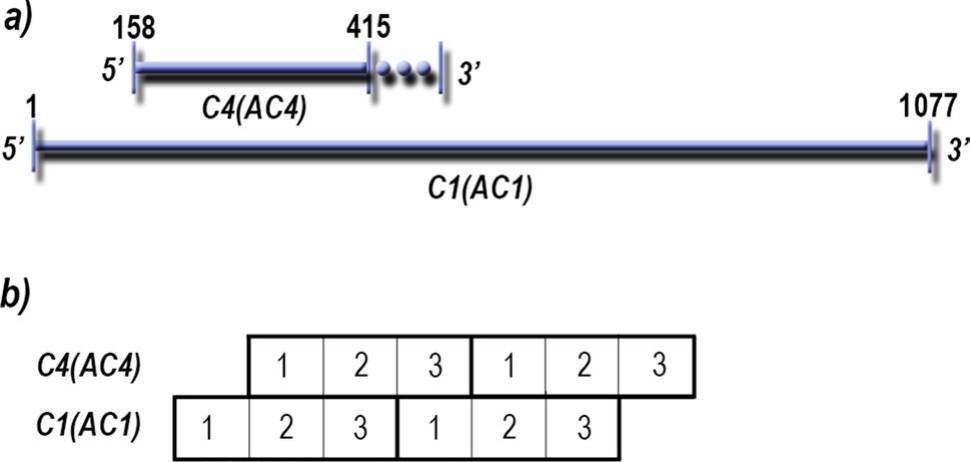
*C4(AC4)* overprinted on *C1(AC1).* (**a**) *C4(AC4)* gene overprinted on the *C1(AC1)* gene. Nucleotide positions refer to nucleotide positions of isolate AF379637 (Beet curly top virus, BCTV-CA/Logan[US:Log:76]) where the BCTV *C4* gene encodes for a protein of 85 amino acids. The region following nucleotide position 415 indicates that some geminiviruses have *C4(AC4)* genes that encode for proteins that have lengths greater than 85 amino acids. (**b**) Schematic showing + 1 frameshift of *C4(AC4)* relative to the *C1(AC1)*.

*Geminiviridae* is the largest family of plant viruses and contains more species than any other virus family^1^. Geminiviruses cause diseases in a wide range of cultivated and wild hosts worldwide^2^. In cultivated crops these diseases result in significant economic losses and routinely jeopardize food security^3^. Why this virus family, particularly the genus *Begomovirus,* is so diverse is not well-understood. Geminiviruses have small mono- or bipartite circular ssDNA genomes (~ 2.5–5.2 kb) and encode for 4–8 proteins^4^. Four of the nine genera, *Curtovirus* (three species), *Topocuvirus* (one species), *Turncurtovirus* (three species) and *Begomovirus* (> 420 species) contain a small gene, designated *C4* (*AC4* in bipartite begomoviruses), that is nested within the *C1* gene (*AC1* in bipartite begomoviruses)^4^. *C4(AC4)* genes encode for, or have the potential to encode for, a protein of approximately 10 kDa^4^. *C4(AC4)* likely originated by the mechanism of overprinting within *C1(AC1)* (Fig. 1a), which occurs when mutations result in the generation of a de novo gene that overlaps an existing gene. Generally, overprinting occurs through a + 1 frameshift^5,6^, which is the case with *C4*(*AC4)* (Fig. 1b).

Gene overprinting allows for viruses to increase coding capacity, and subsequently genetic variability, while maintaining small, compact genomes. In viruses, overlapping genes may experience different forms of selection, including being under evolutionary constraint^7,8^, being under differing evolutionary constraints with the de novo gene usually being under positive or weak purifying selection and the ancestral gene being under purifying selection^6^, or being under independent adaptive evolution^9^. The viral proteins encoded by many overprinted de novo genes are predicted to be intrinsically disordered and are commonly accessory proteins affecting pathogenicity or viral movement rather than virus replication or structure^5^, which is in line with the presently known functions of C4(AC4).

The *C1(AC1)* gene is conserved in genome location and function throughout the *Geminiviridae* family^4^, whereas *C4(AC4)* genes are present in only 4 geminivirus genera, so we assume for further discussion that *C1(AC1)* represents the ancestral gene. *C1(AC1)* encodes a multifunctional replication-associated protein (Rep), which is the only geminivirus protein essential for replication and, therefore, likely to be under strong purifying selection. In addition, C1(AC1) proteins, which do not have polymerase activity, restore DNA replication competency to terminally differentiated host cells establishing an environment for virus replication^10^. The *C4(AC4)* gene is overprinted on a portion of the 5′-half of the *C1(AC1)* gene that encodes for domains involved in DNA binding and cleavage/ligation, oligomerization, and in interacting with multiple proteins^11^.

Available information on the role of the C4(AC4) proteins from isolates of the *Curtovirus* and *Begomovirus* genera suggests a variety of functions, which likely indicate the evolving nature of the proteins. Known functions within the curtovirus C4 proteins include the induction of hyperplasia^12–16^ and a role in systemic movement^17^. Functions within monopartite begomovirus C4 proteins include compromising the hypersensitive response^18^, enhancing drought tolerance^19^, enhancing virus accumulation^20^, induction of hyperplasia^20^, movement^21–23^, suppression of the salicylic acid defense response^24^, and suppression of RNA silencing^20,25–30^. Functions within bipartite begomovirus C4 proteins include induction of hyperplasia^31^, maintenance of a betasatellite^32^, and suppression of RNA silencing^33–36^. In some cases, bipartite begomovirus C4(AC4) proteins have no detectable functions^37–40^. Many of the C4(AC4) functions likely arise from multiple C4(AC4)-host protein interactions, including Shaggy-like protein kinases^16,31,41^, Clavata1-type plasma membrane receptor-like kinases^25,33,42^, S-adenosyl Methionine Synthetase^26^, and Hypersensitive Induced Reaction 1^18^. Some AC4 related functions result from the protein binding to miRNAs and siRNAs and acting as a suppressor of RNA-silencing^28,36^. The function of the C4 proteins encoded in the *Topocuvirus* and *Turncurtovirus* genera have not been studied to date.

Limited information is available on genetic diversity within the *C4(AC4)* genes. Previous studies were restricted to natural populations of single geminivirus species and were limited in their inferences. An analysis of natural populations on a local scale of Tobacco leaf curl virus (21 isolates; this virus is likely referred to now as Eupatorium yellow vein virus) from *Eupatorium makinoi*^43^ or begomoviruses causing cotton leaf curl disease (14 isolates) from cotton^7^ suggest that *C4* is evolutionarily constrained and conserved through purifying selection. A study of Tomato yellow leaf curl China virus (TYLCCNV) within a naturally infected tomato (*Solanum lycopersicum*) plant, or *Nicotiana benthamiana* and tomato plants inoculated with a DNA clone of TYLCCNV, reported that a higher mutational rate occurs in the *AC1-AC4* overlapping region than the upstream non-overlapping region of *AC1*^44^. More recently, research on the genetic diversity of the *C4* and *C1* overlapping sequences from 11 isolates of Tomato leaf deformation virus^45^ indicated that the *C4* gene sequence was under positive selection and corresponding *C1* gene sequence was under purifying selection.

To gain a broader and more extensive understanding of the evolution of a diverse group of geminivirus *C4(AC4)* gene sequences relative to the *C1(AC1)* gene sequences that they overlap, the ratios of the rates of nonsynonymous (*d*_N_) to synonymous (*d*_S_) nucleotide substitutions (ω = *d*_N_/*d*_S_) were determined for each coding region from isolates representing 200 species of begomoviruses and from 11 isolates of a single curtovirus species. We also evaluated *C4(AC4)* diversity within a group of begomoviruses isolated from tomato in native and exotic locations of the host. Last, we compare the intrinsically disordered nature of the geminivirus C4(AC4) proteins to the amino acid sequence from the overprinted C1(AC1) region to evaluate the potential for structural-disorder derived functional diversify in C4(AC4) that might explain how the protein can obtain varying roles. Our analyses suggest that begomovirus and curtovirus C4(AC4) proteins are rapidly evolving through strong positive selection and that the intrinsically disordered nature of C4/AC4 proteins could generate multifunctional characteristics.

## Results

### Selection of geminivirus *C4(AC4)* and *C1(AC1)* gene sequences

*N*-terminal myristoylation of C4(AC4) proteins from the *Curtovirus* and *Begomovirus* genera has been shown to be necessary for function^16,31,34^. Therefore, all *C4(AC4)* gene sequences analyzed begin with the ATG codon that immediately precedes a *N*-terminal myristoylation motif. All *C4(AC4)* genes are in a + 1-frameshift relative to the *C1(AC1)* reading frame, so the *C1(AC1)* coding sequences analyzed begin at the third nucleotide of the *C4(AC4)* gene, to retain the *C1(AC1)* reading frame. The *Begomovirus* genus is composed of > 420 species^4^. We randomly selected 200 species for analysis, using the exemplary isolate from each species^4^. Isolates analyzed from the *Begomovirus* genus fall into six groups, C4(AC4) proteins composed of 85, 90, 94, 96, 97 or 100 amino acids in length (Supplementary Table S1). All *C4* genes in isolates from the *Curtovirus, Topocuvirus* and *Turncurtovirus* genera encode proteins of 85 amino acids in length (255 nucleotides). The *Curtovirus* genus is composed of three species, with only the Beet curly top virus (BCTV) species having a *N*-terminal myristoylation motif in most of its isolates. Eleven isolates from the BCTV species were analyzed. Two *Curtovirus* species, Horseradish curly top virus and Spinach severe curly top virus, have single isolates that do not have a *N*-terminal myristoylation motif and were not included in the analysis. From the *Turncurtovirus* genus, four isolates of the Turnip curly top virus species and the single isolate of the Turnip leaf roll virus species were included in the maximum likelihood (ML) phylogenetic analysis and the intrinsically disordered protein analysis, as was, Tomato pseudo-curly top virus, the single isolate in the *Topocuvirus* genus. BCTV coat protein (CP) gene sequences (762 nucleotides), which are conserved^7,46,47^, were used as a control for evaluating purifying selection. Stop codons were not included in the analyses.

### *C4(AC4)* genes are under positive selection

**Figure 2:**
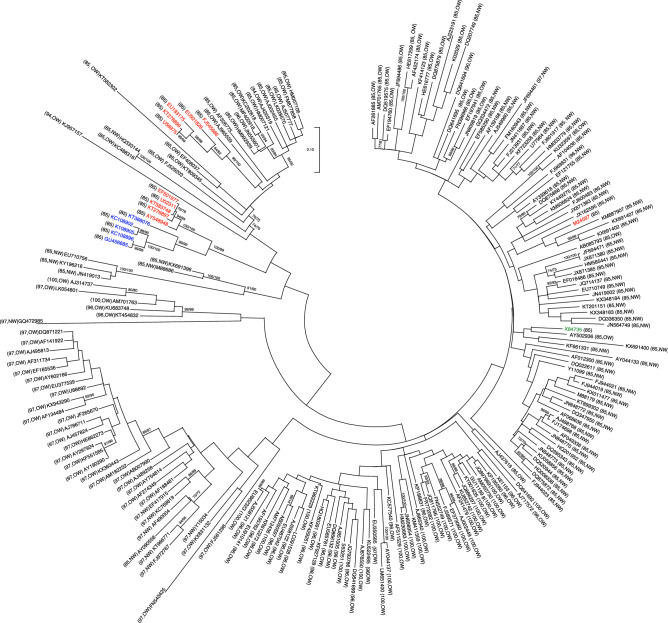
Maximum likelihood (ML) phylogenetic tree of *C4(AC4)* sequences of diverse geminivirus isolates. ML tree based on a Hasegawa, Kishino, and Yano model of evolution assuming a gamma distribution and invariable sites (HKY + G + I) showing genetic relationships among *C4(AC4)* sequences (nucleotides 1–255) from four genera of the family *Geminiviridae*; exemplary isolates from 200 species of the *Begomovirus* genus, 11 isolates from the Beet curly top virus species of the *Curtovirus* genus, four isolates of the Turnip curly top virus species and the single isolate of the Turnip leaf roll virus species from the *Turncurtovirus* genus, and Tomato pseudo-curly top virus, the single isolate in the *Topocuvirus* genus. The ML tree for *C1(AC1)* sequences (255 nucleotides) that the *C4(AC4)* genes overlap is highly identical and not shown. Gene sequences are taken from full-length genomic sequences and the GenBank accession number of each virus is indicated. Preceding or following the GenBank accession numbers in parentheses are the amino acid length of the respective C4(AC4) proteins and the designation for New World (NW) or Old World (OW). Numbers at branches represent bootstrap values (≥ 70% are shown) determined from 500 replicates. Since the ML trees for *C4(AC4)* and *C1(AC1)* are nearly identical, bootstrap values for *C4(AC4)* and *C1(AC1)* are separated by a slash. The bar near the top of the cladogram represents nucleotide substitutions per site for the *C4(AC4)* sequences. Virus isolate(s) in black text are begomoviruses, red text are curtoviruses, blue text are turncurtoviruses, green text is a topocuvirus.

We initially looked at the evolutionary relationships among *C4(AC4)* genes, as well as the overprinted *C1(AC1)* gene sequences. A phylogenetic tree (Fig. 2) was inferred using maximum likelihood in MEGAX^48^. The inferred phylogeny of *Geminiviridae* members based on *C4(AC4)* and *C1(AC1)* sequences had little to no strong statistical support as many of the terminal nodes and nearly all of the basal nodes had uniformly low statistical support (bootstrap support values < 0.5). While the phylogeny is poorly resolved, the lack of resolution and lack of strong node support suggests that the *C4(AC4)* and *C1(AC1)* genes accrue a high number of mutations relatively quickly, which obscures the phylogenetic signal from lineages with shared descent. Interestingly, 1 BCTV isolate (M24597, BCTV/CA/Logan) did not group with the other 10 BCTV isolates, suggesting this isolate has undergone recombination.

To determine if *C4(AC4)* is under positive selection we analyzed nucleotide substitutions in *C4(AC4)* compared to substitutions in the same portion of the *C1(AC1)* sequence that *C4(AC4)* overlaps for a diverse group of *Begomovirus* and *Curtovirus* isolates (Fig. 2). For the 200 *Begomovirus C4(AC4)* gene sequences, ω (the ratio of the number of nonsynonymous substitutions per nonsynonymous site to the number of synonymous substitutions per synonymous site) was 1.528 for the initial 255 nucleotides representing 85 amino acids, indicating that *C4(AC4)* is under strong positive selection (Table 1). Likelihood ratio tests (LRT) applied to the three pairs of models in CODEML indicated that ω varied across codons (M0 vs. M3) and that *C4(AC4)* was under positive selection (M2a vs. M1a and M8 vs. M7) (Supplementary Table S2). The Bayes empirical Bayes (BEB) analysis showed that 23 codon sites were under positive selection with a posterior probability (PP) of ≥ 95% (Table 1), which represents approximately 27% of the amino acid residues encoded by the *C4(AC4)* gene. Additionally, the codons under positive selection were not restricted to specific regions of the gene, but were spread across the entire length of *C4(AC4)*, suggesting that mutations across the entire gene sequence generates functional protein diversity (*e.g.* absence of premature stop codons). In contrast, the length of the *C1* sequences that the *C4(AC4)* is overprinted on were under purifying selection (ω = 0.127; Table 1). Although ω varied across codons (M0 vs. M3), the overprinted region of *C1* was not under positive or neutral selection (M2a vs. M1a and M8 vs. M7) (Supplementary Table S2), nor were any individual codons suggested to be under positive selection (BEB) (Table 1).

**Table 1:** Positive selection analyses of *C4(AC4)* or *C1(AC1)* gene sequences from begomovirus and curtovirus species.

Coding region	Nucleotides in coding region	Amino acids	ω^a^	Positively selected sites^b^
**Gene sequences of 200 begomoviruses**				
*C4/AC4*	255	85	1.528	30 (**23**)^c^ **3S**, 6S, **8C**, **9L**, **10F**, 11S, **14E**, **16T**, **17T**, **19K**, **21N**, **27Y**, **29Q**, **30P**, **31G**, **37Q**, **43Q**, **44A**, 52T, **57P**, 58L, **62N**, **67E**, 73A, **74A**, **75R**, **76T**, 80L, 82Q, **84P**
*C1/AC1*	255	85	0.127	–
**Gene sequences of 91 begomovirus extensions**				
*C4/AC4*	33	11	0.935	3 (**1**)^d^ 4A, 6S, **7L**
*C1/AC1*	33	11	0.321	–
**Gene sequences of 11 curtovirus isolates**				
*C4*	255	85	1.143	23 (**3**)^e^ 4L, 9C, 16F, 17R, 19Q, 20I, 23Y, 26W, 27Y, **29Q**, 30P, **31G**, 33H, 34I, 37Q, 45A, 50P, **53T**, 68V, 77Q, 81H, 82M, 85R
*C1*	255	85	0.194	–
*CP*	762	254	0.041	–

^a^Mean ratio of the nonsynonymous (*d*_N_) and the synonymous substitution rates (*d*_S_). ω < 1 represents purifying selection; ω = 1 neutral selection; and ω > 1 positive selection.

^b^Number of amino acid sites under positive selection based on Bayes empirical Bayes analysis. Amino acid residue number and single letter amino acid designation having a posterior probability of P > 90% in regular font and of P ≥ 95% in bold font.

^c^Amino acid positions and amino acids refer to HE616777 (African cassava mosaic Burkina Faso virus; ACMBFV).

^d^Amino acid positions and amino acids refer to AJ851005 (Ageratum leaf curl virus; ALCuV).

^e^Amino acid positions and amino acids refer to M24597 (Beet curly top virus; BCTV/CA/Logan).

It was previously suggested that coat protein genes from New World (NW) begomoviruses are under stronger purifying selection than those from Old World (OW) begomoviruses^49^. To determine if NW *C4(AC4)* genes are under lower positive selection than OW *C4(AC4)* genes, we compared the ω-values of *C4(AC4)* from NW versus OW begomoviruses. Of the 200 begomoviuses we analyzed above, 79 were NW viruses and 121 were OW viruses (Fig. 2). The ω-values were 1.545 and 1.550 for the initial 255 nucleotides of the 79 NW *C4(AC4)* genes and the 121 OW *C4(AC4)* genes, respectively. This suggests that *C4(AC4)* genes from NW and OW viruses are under near identical levels of strong positive selection. In contrast, the ω-values of the *C1(AC1)* gene sequences from NW and OW begomoviruses that the *C4(AC4)* genes are overprinted on were under near identical levels of purifying selection (ω = 0.132 and ω = 0.131, respectively).

Ninety-one of the 200 begomoviruses analyzed have C4(AC4) proteins of 96–100 amino acids in length (Supplementary Table S1). An analysis of the extended regions (nucleotides 256–288, representing 11 amino acids) indicated that they were under neutral selection (ω = 0.935) (Table 1, Supplementary Table S2). BEB analysis indicated that 1 of the 11 amino acids were under positive selection with a posterior probability (PP) of ≥ 95%. In contrast, the extended regions represented by the *C1(AC1)* sequences were under purifying selection (ω = 0.321). (Table 1). Of the 91 begomovirus, 83 were OW, suggesting that extended *C4(AC4)* genes are much more common in OW begomoviruses than in NW begomoviruses.

When we analyzed the begomovirus *C4* sequences from 11 isolates from the BCTV species (Fig. 2), the *C4* sequences were also under positive selection (ω = 1.143), while the overlapped *C1* sequences were under purifying selection (ω = 0.194) (Table 1 and Supplementary Table S2). BEB analysis indicated only 3 codons were under strong positive selection (PP ≥ 95%) in the BCTV *C4* genes analyzed (Table 1). As an additional test of purifying selection at a region outside of the *C1* sequences, we determined ω for the coat protein (CP) genes of the 11 BCTV isolates. As expected, the BCTV CP genes, which are highly conserved, were under strong purifying selection (ω = 0.041; Table 1), considerably more so than the *C1* sequences.

To look at variation within site positions that lead to amino acid changes, the relative nucleotide substitution rates within the first, second and third codon positions were determined (Fig. 1b and Table 2). The most variable codon position (highest relative substitution rate) for all overlapping sequences analyzed was the second position of *C4,* which corresponds to the third position of *C1* [C4(AC4)-2/C1(AC1)-3]. To estimate the uniformity of variation at codons, the α parameter was determined as a function of the discrete gamma distribution for each overlapping region^50^ (Table 2). Begomovirus codon position C4(AC4)-2/C1(AC1)-3 (in nucleotides 1–255) displayed an α-value of 1.57. An α > 1 with a high relative substitution rate compared to the other positions indicates that C4(AC4)-2/C1(AC1)-3 positions have intermediate substitution rates, while a few codons have very high or very low relative substitution rates^50^. The high substitution rate and infinitely large α-value for the curtovirus codon positions C4-2/C1-3 indicates a constant substitution rate for all sites. In contrast, codon positions C4(AC4)-2/C1(AC1)-3 for the begomovirus 33 nucleotide extension, had a very high relative substitution rate compared to the other codon positions, but had an α-value of 0.67. An α ≤ 1 indicates that most of the codon positions have very low substitution rates with some sites being hotspots with very high substitution rates^50^.

**Table 2:** Codon-site nucleotide variation in *C4(AC4)* and *C1(AC1)* overlapping regions.

Length (nucleotides)	Codon position	Relative rate	α parameter
**Gene sequences from 200 begomovirus species**			
255	C4(AC4)-1/C1(AC1)-2	1^a^	0.49
255	C4(AC4)-2/C1(AC1)-3	2.41	1.57
255	C4(AC4)-3/C1(AC1)-1	1.19	0.48
**Gene extension sequences from 91 begomovirus species**			
33	C4(AC4)-1/C1(AC1)-2	1^a^	0.30
33	C4(AC4)-2/C1(AC1)-3	6.17	0.67
33	C4(AC4)-3/C1(AC1)-1	2.67	0.65
**Gene sequences from 11 curtovirus isolates**			
255	C4-1/C1-2	1^a^	0.36
255	C4-2/C1-3	2.75	$\infty$
255	C4-3/C1-1	1.66	0.72

^a^The estimated substitution rates are determined relative to this position. For codon position, refer to Fig. 1b.

### Selective pressure on begomoviruses from tomato from native and exotic locations

The movement of crops around the world results in plants encountering new pathogens. This ‘new encounter phenomenon’ occurs when a crop has been introduced into a new geographical region and pests and/or pathogens that evolved with native host species in the introduced region infect and cause disease in the newly introduced exotic crop species^51^. In this study, the native range of a plant is defined as the geographic region in which the species originated as well as the surrounding area where indigenous people may have moved and domesticated the species. Exotic is defined as regions far enough outside the native range of the plant that indigenous people were not likely involved in moving the species, rather it was introduced by movement over long distances. When we tallied whether the begomovirus isolates of > 420 species originated from a host plant within its native range or outside of its native range (exotic) we found that 52.5% of the *Begomovirus* species were isolated from a host plant outside of its native range (Supplementary Table S3).

A large number of *Begomovirus* species (112 of 420) have been isolated from tomato with approximately 70% of the species being isolated from tomato grown outside of western South America and Central America, the native range of tomato and areas where it was likely spread by indigenous people. Ninety-two of the 112 species (29 of the 34 native and 63 of the 78 exotic) had *C4(AC4)* genes with a N-terminal myristoylation motif and a length of 85–100 amino acids. If begomovirus isolates travelled with tomato plants or seed when they were moved from the New World, we might expect to see evidence of purifying selection in *C4(AC4)* if a host-specific interaction would be essential for begomoviruses on tomato outside of its native range (*e.g.* no reservoir of congeners for viruses to move from onto tomato). *C4(AC4)* gene sequences from begomovirus isolates from tomato in native (29 begomoviruses) and exotic (63 begomoviruses) locations (Supplementary Table S4) were under strong positive selection with ω-values of 1.717 and 1.359, respectively, for the initial 255 nucleotides representing 85 amino acids (Table 3). Likelihood ratio tests (LRT) applied to the three pairs of models in CODEML indicated that ω varied across codons (M0 vs. M3) and that *C4(AC4)* was under positive selection (M2a vs. M1a and M8 vs. M7) (Supplementary Table S5). The codons under positive selection were located throughout the length of the *C4(AC4)* reference sequence, suggesting changes across the entire gene sequence may generate protein diversity. As expected, the *C1(AC1)* sequences were under purifying selection (Table 3 and Supplementary S5). Therefore, there is no evidence of either purifying or neutral selection in *C4(AC4)* genes from tomato-associated isolates taken from native or exotic regions.

**Table 3:** Positive selection analyses of *C4(AC4*) or *C1(AC1)* gene sequences from begomovirus species isolated from *Solanum lycopersicum* from native and non-native locations.

Coding region	Nucleotides in coding region	Amino acids	ω^a^	Positively selected sites^b^
**Gene sequences of 29 species from native locations**				
*C4/AC4*	255	85	1.717	29 (**19**)^c^ **8F**, **9S**, **10S**, 11N, **14G**, 15S, 16T, **17S**, **19R**, **21T**, **27S**, 31G, 37R, 39Y, **43P**, **44A**, **48S**, 49P, **57Q**, **58L**, 62N, **67A**, 70L, **74S**, **75N**, **76L**, 77L, **82P**, **84R**
*C1/AC1*	255	85	0.134	2 **(1)** 57V, **73Q**
**Gene sequences of 63 species from non-native locations**				
*C4/AC4*	255	85	1.359	28 (**23**)^d^ **3L**, **6S**, **8P**, **9S**, 10S, **11S**, **14V**, **16P**, **17S**, 18S, **19E**, **21P**, **26S**, **27L**, **30I**, **31T**, 37Q, **44P**, **45A**, **49S**, 51T, **53R**, **58T**, **68E**, **74V**, **76R**, 81Q, **85P**
*C1/AC1*	255	85	0.157	3(0) 2S, 16Q, 18R

^a^Mean ratio of the nonsynonymous (*d*_N_) and the synonymous substitution rates (*d*_S_). ω < 1 represents purifying selection; ω = 1 neutral selection; and ω > 1 positive selection.

^b^Number of amino acid sites under positive selection base on Bayes empirical Bayes analysis^47^. Amino acid residue number and single letter amino acid designation having a posterior probability of P > 90% in regular font and of P ≥ 95% in bold font.

^c^Amino acid positions and amino acids refer to AF101476 (Chino del tomate virus; CdTV).

^d^Amino acid positions and amino acids refer to JN135234 (Pepper leaf curl Lahore virus; PepLCLaV).

### Geminivirus *C4(AC4)* genes encode intrinsically disordered proteins

**Figure 3:**
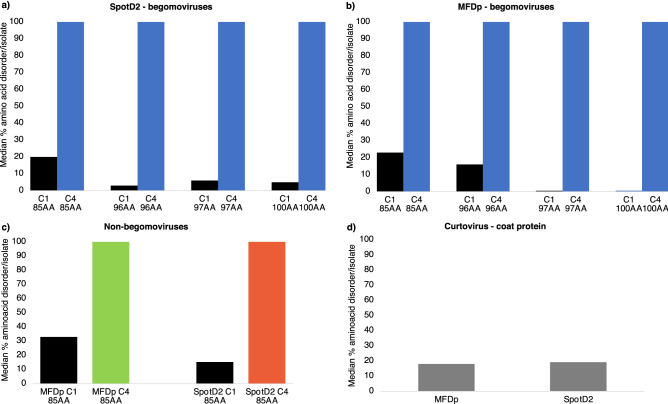
Intrinsically disordered protein analysis. (**a**) Medium percentage amino acid intrinsic disorder/begomovirus isolate determined by SPOT-Disorder2 for C4(AC4) proteins and the overlapped C1(AC1) protein regions. (**b**) Medium percentage amino acid intrinsic disorder/begomovirus isolate determined by MFDp2 for C4(AC4) proteins and the overlapped C1(AC1) protein regions. For (**a**) and (**b**), 106 begomovirus isolates encoded C4(AC4) proteins and the corresponding C1(AC1) regions of 85 amino acids in length, 26 isolates of 96 amino acids in length, 37 isolates of 97 amino acids in length and 28 isolates of 100 amino acids in length (Supplementary Table S6). (**c**) SPOT-Disorder2 and MFDp2 analysis of encoded C4(AC4) proteins and the overlapped C1(AC1) protein regions of non-begomovirus isolates, 85 amino acids in length, that included the isolates from the *Curtovirus*, *Turncurtovirus* and *Topocuvirus* genera (Supplementary Table S6). (**d**) SPOT-Disorder2 and MFDp2 analysis of curtovirus coat proteins, 254 amino acids in length (Supplementary Table S6).

Most overprinted de novo virus genes are predicted to encode intrinsically disordered proteins (IDPs)^5^. The inherent flexability with IDPs is a major factor in the interactions with multiple partners affecting cellular interactions regulating development, metabolic and signaling pathways and stress responses^52,53^. To determine if this was the case with the C4(AC4) proteins, 2 protein disorder prediction programs were utilized. Both disorder predictors gave similar results with all 217 C4(AC4) proteins predicted to be fully disordered (Fig. 3a–c; Supplementary Table S6). The same analysis predicts that overlapping *C1(AC1*) coding regions are primarily ordered. This is especially true for the overlapping *C1(AC1)* regions of begomoviruses *C4(AC4)* gene sequences encoding for proteins of 100 amino acids in length (Fig. 3), which would suggest that within the intact C1(AC1) proteins this region is highly ordered. As a control, the disorder predictors were used to analyze the structure of the curtovirus CP. Since the CPs of icosahedral viruses have a canonical ß-barrel jelly roll structure^54^, we expect the BCTV CPs to be mostly ordered. The N-termini of the BCTV CPs are disordered (~ 18% of the proteins), while the C-terminal (82% of the protein) is predicted to be ordered (Fig. 3d; Supplementary Table S6). While the ordered region includes the ß-barrel jelly roll structure of the CPs, the disordered N-terminal region has been shown to bind DNA and take on order that results in capsid assembly^55,56^. Taken together, the results suggest that C4(AC4) proteins are IDPs while C1(AC1) proteins are mostly ordered.

## Discussion

Overprinted de novo genes in virus genomes are thought to be an evolutionary mechanism to maximize coding potential and to provide genetic novelty. Frequently, young de novo genes are rapidly evolving relative to the ancestral gene, allowing for the evolution and development of diverse novel genetic functions, while older de novo genes may be under purifying selection and evolve more slowly^6^. Although nucleotide variation occurs and is generally assumed to be selected for in the overprinted gene, at least in the + 1 frameshift, the amino acid sequence of the protein encoded by the ancestral gene is conserved due to redundancy in the genetic code. Most of the nucleotide variation occurs in the third position (wobble position, genetic degeneracy allows for a higher probability of a synonymous change) of the codons of the ancestral gene, which is the second codon position in the overprinted gene (+ 1 frameshift) (Fig. 1b). Mutations in codon position 2 that do not result in stop codons are always non-synonomous mutations that lead to an amino acid change. Indeed, the α parameter value indicated that position 3 of *C1(AC1)* codons and position 2 of *C4(AC4)* codons had the highest relative rate of nucleotide substitution and were the most variable. Thus, the overprinted gene, *C4(AC4)*, undergoes positive selection while the ancestral gene, *C1(AC1)*, remains under purifying selection.

The overall mutation rate in the overlapping genes appears to be rapid enough to obscure a clear phylogenetic signal as we were unable to generate a majority resolved phylogeny with statistical confidence. However, purifying selection on *C1(AC1)* likely favors the accumulation of synonymous substitutions while positive selection on *C4(AC4)* favors the relatively rapid accrual of non-synonymous substitutions, and presumably C4(AC4) protein diversity.

Since the *C4(AC4)* genes that have been studied have diverse functions, we determined the type of selective pressure the genes are under relative to the *C1(AC1)* sequences the *C4(AC4)* genes overlap. As a group, the ω-values for the *C4(AC4)* genes of the begomoviruses (ω = 1.528) were under greater positive selection than the *C4* of the curtovirus isolates (ω = 1.143) within nucleotides 1–255. This difference is likely because the curtovirus isolates are from 1 species, while the begomovirus isolates represent 200 species, reflecting greater genetic and taxonomic diversification.

Relative to the positive selection observed for nucleotides 1–255 in begomovirus *C4(AC4)* genes, near neutral selection (ω = 0.935) occurred in the extended region of the *C4(AC4)* genes having protein lengths of 96–100 amino acids. While the extended region is not evolving as rapidly as the region of *C4(AC4)* representing nucleotides 1–255, diversity in the extended region is occurring more rapidly than the overlapped *C1(AC1)* region, which is still under purifying selection (ω = 0.321). The elimination of a stop codon and increase in length of C4(AC4) proteins could provide a domain with novel function or the ability to modulate existing C4(AC4) function. It may be that the extended regions of the begomovirus *C4(AC4)* genes, especially in OW begomoviruses, represent a more recent event that results in more rapid evolution than that observed in the core *C4(AC4)* genes representing the initial 1–255 nucleotides. The generation of a de novo gene by overprinting and protein extension by elimination of stop codons has been proposed for the creation of the ALTO/MT gene in polyomviruses^57^. In the case of the begomoviruses, our study suggests that the entire length of the *C4(AC4)* (nucleotides 1–255) is available for generating protein diversity through strong positive selection as well as the elongation of *C4(AC4)* genes, albeit the extended regions evolve less rapidly. These data support the conclusion that positive selection of *C4(AC4)* genes could explain how the resulting proteins have evolved diversified and diverged functions. In contrast, purifying selection of *C1(AC1)* is consistent with its essential role in virus replication.

All C4(AC4) proteins analyzed were predicted to be IDPs. An important characteristic of IDPs is their ability to be promiscuous, binding multiple partners (i.e., proteins or nucleic acid)^53,58^. While the compact nature of virus genomes restricts the number of virus-encoded proteins, rapidly evolving IDPs would give the virus an advantage in the ability of the IDPs to interact with multiple host targets, resulting in multiple functions. Depending on the environment and binding partners, IDPs take on different conformations and different functions. Inducibility of more ordered structure may also occur through post-translation modifications^53^, such as regulation of BRASSINISTEROID INSENSITIVE I KINASE INHIBITOR in the brassinosteroid pathway by phosphorylation^59,60^. Indeed, phosphorylation of Ser49 in the BCTV C4 protein is required for the protein to bind to and inhibit the function of AtSKs and negatively regulate the brassinosteroid pathway^16^. Therefore, phosphorylation of Ser49 may induce and stabilize a C4 conformation required for function. Since C4(AC4) proteins bind a number of targets, including various host proteins and mi/siRNAs, the intrinsically disordered nature of C4(AC4) may allow the proteins to be multifunctional and the high rate of non-synonymous substitutions would allow the proteins to abruptly modify existing functions or to take on new functions. Because of the high diversity within C4(AC4) proteins, each C4(AC4) might be expected to bind a specific set of host partners and members of these sets might overlap between different C4(AC4) proteins, allowing for some redundancy as well as unique specificities.

Our results indicate that curtovirus and begomovirus *C4(AC4)* genes encode for rapidly evolving IDPs. Typically, overprinted de novo genes encode for accessory proteins that contribute to viral pathogenicity, but are not structural proteins and are unnecessary for virus replication. Begomovirus *C4(AC4)* genes have been implicated in modulating numerous host defense reactions^18,20,24–30,33–36^. It is noteworthy that positive selection has been shown to drive rapid evolution of antiviral RNAi genes in *Drosophila* and multiple invertebrates^61,62^. Therefore, it might be inferred that rapid evolution of begomovirus *C4(AC4)* genes could be reflective of the proteins adapting to rapidly evolving antiviral RNAi host genes in plants. Indeed, plant virus suppressors of RNAi have been shown to be subject to positive selection that could be attributed to frequent shifts between host species^63^.

*Begomovirus* is the largest virus genus with diversification primarily driven by mutational dynamics and substitution rates similar to RNA viruses^47,64^. Over half of the recorded *Begomovirus* species were isolated from plants that occurred in biogeographic regions outside of their native range (Table Supplementary S3). Tomato-associated begomoviruses represent a large group of viruses isolated from the host’s native and exotic locations. Positive selection and the gene-wide distribution of positively selected codon positions on the *C4(AC4)* gene was comparable in tomato-associated *Begomovirus* regardless of geographic range of the tomato host. Furthermore, these same patterns of positive selection on *C4(AC4)* were observed throughout all the begomoviruses in this study. Because *C4(AC4)* has a diversity of known functions, including a role in host infection^65,66^, it is tempting to speculate that positive, diversifying selection over the whole *C4(AC4)* gene, coupled with intrinsically disordered nature of C4(AC4) could provide begomoviruses the innate flexibility to more rapidly adopt to new hosts and radiate where they are found anywhere in the world.

Lastly, it is worth noting that geminivirus sequences in GenBank are biased towards crop plant hosts. Therefore, it is not unreasonable to predict that *C4(AC4)* variability may be greater, considering the number of begomovirus species is likely larger than those presently characterized when also considering geminiviruses yet to be identified from non-crop species worldwide. Future experiments will provide additional insights and clarity into the mechanism(s) by which C4(AC4) proteins function and will aid in determining the extent to which positive selective pressure and intrinsic disorder diversifies C4(AC4) function.

## Methods and materials

### Selection of geminivirus gene sequences

All sequences analyzed were obtained from full-length geminivirus genomic sequences available in GenBank (GenBank accession numbers are given in Fig. 2). All begomovirus sequences used to determine the ratios of the rates of non-synonymous (*d*_N_) to synonomous (*d*_S_) nucleotide substitutions (ω = *d*_N_/*d*_S_) were randomly selected from listed species available in the International Committee on Taxonomy of Viruses (ICTV) Report on the taxonomy of the *Geminiviridae*^4^ (Available online at https://www.ictv.global/report/geminiviridae).

### Non-synonymous-synonymous substitution rate and codon site nucleotide variation

Phylogenetic trees were inferred using maximum likelihood (ML) in MEGAX^48^. Evolutionary models of nucleotide substitution were determined based on ML in MEGAX. Initially, *ω* was calculated based on one ratio (M0) with the maximum likelihood method by CODEML in PAML v4.8 package^67^ contingent on nucleotide alignments and neighbor-joining trees based on the Jukes-Cantor model constructed in Geneious v6^68^. To detect the statistical power of positive selection and the individual amino acid residues under positive selection, likelihood ratio tests were applied on three pairs of models in the program CODEML to test for departure from neutral models: one ratio (M0) vs. discrete (M3), nearly neutral (M1a) vs. positive selection (M2a), and *β* (M7) vs. *β* & *ω* (M8). To determine if non-neutral models best fit the data, we compared twice the difference of the log-likelihood values of each pair of models using a χ^2^ distribution. Amino acid sites under positive selection were estimated by Bayes empirical Bayes analysis based on the M8 model^69^. Codon substitution rates and α parameters were determined using a maximum likelihood approach in the module BASEML within PAML^67–69^.

### Predicting intrinsically disordered

There are numerous computational software tools available for predicting intrinsically disordered protein regions. Based on different methodologies available, SPOT-Disorder2 and MFDp2 were used to determine intrinsic disorder in C4(AC4) and C1(AC1) amino acid sequences from 217 geminivirus isolates. SPOT-disorder2 is an updated version of a highly rated disorder predictor that uses an ensemble of deep bidirectional long short-term memory and inception-residual squeeze-and excitation convolutional neural network^70–72^. MFPp2 is a highly rated meta-disorder predictor, which combines the methodology of a number of predictors^72,73^.

## Supplementary Information

Supplementary Table S1.

Supplementary Table S2.

Supplementary Table S3.

Supplementary Table S4.

Supplementary Table S5.

Supplementary Table S6.
