# Supplementary material for: Positive selection and intrinsic disorder are associated with multifunctional C4(AC4) proteins and geminivirus diversification
Source: Sci Rep. 2021 May 27;11:11150. doi: 10.1038/s41598-021-90557-0 (PMC8160170; doi:10.1038/s41598-021-90557-0)
Supplement: Supplementary file 1 — Supplementary Table S1. [file 41598_2021_90557_MOESM1_ESM.docx]

| Supplementary Table S1. Number of amino acids encoded in the 200 begomovirus *C4/AC4* genes analysed. | |
| --- | --- |
| Amino acid length  of C4/AC4 | Number of begomovirus  species (%) |
| 85 | 106 (53) |
| 90 | 1 (-) |
| 94 | 2 (1) |
| 96 | 26 (13) |
| 97 | 37 (19) |
| 100 | 28 (14) |
