# Supplementary material for: Positive selection and intrinsic disorder are associated with multifunctional C4(AC4) proteins and geminivirus diversification
Source: Sci Rep. 2021 May 27;11:11150. doi: 10.1038/s41598-021-90557-0 (PMC8160170; doi:10.1038/s41598-021-90557-0)
Supplement: Supplementary file 2 — Supplementary Table S2. [file 41598_2021_90557_MOESM2_ESM.docx]

Supplementary Table S2. To detect the statistical power of positive selection, likelihood ratio tests were applied on three pairs of models: one ratio

(M0) *vs*. discrete (M3), positive selection (2a) *vs.* nearly neutral (M1a), and β (M7) *vs.* β & ω (M8).

|  | M0 M3 2ΔL ρ-value | M1a M2a 2ΔL ρ-value | M7 M8 2ΔL ρ-value |
| --- | --- | --- | --- |
|  | | | |
| Gene sequences from 200 species of begomoviruses | | | |
| *C4(AC4)* (255)  *C1(AC1)* (255) | -18856.08 -18268.35 1175.46 <0.001  -17355.78 -16511.24 1689.08 <0.001 | -18644.74 -18294.53 700.42 <0.001  -16945.45 -16945.45 0 1.000 | -18602.61 -18273.93 657.36 <0.001  -16483.43 -16484.52 2.18 0.336 |
| Gene sequence from 91 begomovirus extensions | |  |  |
| *C4(AC4)* (33)  *C1(AC1)* (33) | -1004.56 -990.71 27.70 <0.001  -685.68 -659.95 51.46 <0.001 | -997.55 -992.39 10.32 0.006  -673.31 -673.31 0 1.000 | -996.29 -991.18 10.22 0.006  -662.24 -666.74 9.00 0.011 |
| Gene sequences from 11 curtoviruses | |  |  |
| *C4* (255)  *C1* (255) | -1109.65 -1101.03 17.24 0.002  -1060.42 -1028.90 63.04 <0.001 | -1107.35 -1101.68 11.34 0.003  -1033.38 -1033.38 0 1.000 | -1107.86 -1101.69 12.34 0.002  -1032.43 -1029.51 5.84 0.053 |
| *CP* (762) | -2724.70 -2664.10 121.20 <0.001 | -2686.65 -2686.65 0 1.000 | -2664.82 -2664.75 0.14 0.932 |
|  | | | |
