# Supplementary material for: Positive selection and intrinsic disorder are associated with multifunctional C4(AC4) proteins and geminivirus diversification
Source: Sci Rep. 2021 May 27;11:11150. doi: 10.1038/s41598-021-90557-0 (PMC8160170; doi:10.1038/s41598-021-90557-0)
Supplement: Supplementary file 3 — Supplementary Table S3. [file 41598_2021_90557_MOESM3_ESM.docx]

| Supplementary Table S3. Summary of *Begomovirus* species isolated from a host plant exotic to the geographic region of isolation. | | |
| --- | --- | --- |
| Host plant Order | Number of different *Begomovirus* spp. | % *Begomovirus* spp. isolated from an exotic host plant |
| Solanales (2^a^) | 156 | 69.5% |
| Malvales (1) | 83 | 36.8% |
| Fabales (1) | 45 | 24.4% |
| Malpighiales (3) | 31 | 82.6% |
| Asterales (1) | 20 | 50.0% |
| Lamiales (5) | 20 | 40.0% |
| Cucurbitales (1) | 15 | 66.7% |
| Brassicales (3) | 8 | 100% |
| Gentianales (3) | 6 | 66.7% |
| Rosales (2) | 6 | 0% |
| Caryophyllales (2) | 4 | 25% |
| Myrtales (1) | 3 | 66.7% |
| Dipsicales (1) | 1 | 100% |
| Oxalidales (1) | 1 | 100% |
| Sapindales (1) | 1 | 0% |
| Total | | |
| 15 Orders (28 families) | 400 *Begomovirus* spp. | 52.5% |

**^a^** Number in parentheses is the number of Families containing begomoviruses in

each Order
