# Supplementary material for: Positive selection and intrinsic disorder are associated with multifunctional C4(AC4) proteins and geminivirus diversification
Source: Sci Rep. 2021 May 27;11:11150. doi: 10.1038/s41598-021-90557-0 (PMC8160170; doi:10.1038/s41598-021-90557-0)
Supplement: Supplementary file 5 — Supplementary Table S5. [file 41598_2021_90557_MOESM5_ESM.docx]

Supplementary Table S5. To detect the statistical power of positive selection, likelihood ratio tests were applied on three pairs of models: one ratio

(M0) *vs*. discrete (M3), positive selection (2a) *vs.* nearly neutral (M1a), and β (M7) *vs.* β & ω (M8).

|  | M0 M3 2ΔL ρ-value | M1a M2a 2ΔL ρ-value | M7 M8 2ΔL ρ-value |
| --- | --- | --- | --- |
| Gene sequences from 29 tomato isolates of begomoviruses from native regions | | | |
| *C4(AC4)* (255)  *C1(AC1)* (255) | -3539.16 -3422.71 232.90 <0.001  -3292.17 -3148.98 286.38 <0.001 | -3510.30 -3425.93 168.74 <0.001  -3193.89 -3191.78 4.22 0.121 | -3515.69 -3425.94 179.50 <0.001  -3150.32 -3143.57 13.50 0.001 |
|  | | | |
| Gene sequences from 63 tomato isolates of begomoviruses from exotic regions | | | |
| *C4(AC4)* (255)  *C1(AC1)* (255) | -5831.57 -5676.80 309.54 <0.001  -5418.49 -5132.65 571.68 <0.001 | -5758.06 -5684.92 146.28 <0.001  -5216.62 -5215.72 1.80 0.407 | -5765.43 -5685.63 159.60 <0.001  -5126.92 -5127.40 0.96 0.619 |
